# Health risk assessment of China’s main air pollutants

**DOI:** 10.1186/s12889-017-4130-1

**Published:** 2017-02-20

**Authors:** Jian Sun, Tiancai Zhou

**Affiliations:** 1 Institute of Geographic Sciences and Natural Resources Research, Chinese Academy of Sciences, 11A, Datun Road, Chaoyang District, Beijing, 100101 China; 2 Chengdu University of Technology, Chengdu, 610000 China

**Keywords:** Air pollutants, Haze, Spatial patterns, Health risk, China

## Abstract

**Background:**

With the rapid development of China’s economy, air pollution has attracted public concern because of its harmful effects on health.

**Methods:**

The source apportioning of air pollution, the spatial distribution characteristics, and the relationship between atmospheric contamination, and the risk of exposure were explored. The in situ daily concentrations of the principal air pollutants (PM_2.5_, PM_10_, SO_2_, NO_2_, CO and O_3_) were obtained from 188 main cities with many continuous air-monitoring stations across China (2014 and 2015).

**Results:**

The results indicate positive correlations between PM_2.5_ and SO_2_ (*R* ^2^ = 0.395/0.404, *P* < 0.0001), CO (*R* ^2^ = 0.187/0.365, *P* < 0.0001), and NO_2_ (*R* ^2^ = 0.447/0.533, *P* < 0.0001), but weak correlations with O_3_ (*P* > 0.05) for both 2014 and 2015. Additionally, a significant relationship between SO_2_, NO_2,_ and CO was discovered using regression analysis (*P* < 0.0001), indicating that the origin of air pollutants is likely to be vehicle exhaust, coal consumption, and biomass open-burning. For the spatial pattern of air pollutants, we found that the highest concentration of SO_2_, NO_2,_ and CO were mainly distributed in north China (Beijing-Tianjin-Hebei regions), Shandong, Shanxi and Henan provinces, part of Xinjiang and central Inner Mongolia (2014 and 2015).

**Conclusions:**

The highest concentration and risk of PM_2.5_ was observed in the Beijing–Tianjin–Hebei economic belts, and Shandong, Henan, Shanxi, Hubei and Anhui provinces. Nevertheless, the highest concentration of O_3_ was irregularly distributed in most areas of China. A high-risk distribution of PM_10_, SO_2_ and NO_2_ was also observed in these regions, with the high risk of PM_10_ and NO_2_ observed in the Hebei and Shandong province, and high-risk of PM_10_ in Urumchi. The high-risk of NO_2_ distributed in Beijing-Yangtze River Delta region-Pearl River Delta region-central. Although atmospheric contamination slightly improved in 2015 compared to 2014, humanity faces the challenge of reducing the environmental and public health effects of air pollution by altering the present mode of growth to achieve sustainable social and economic development.

## Background

Haze is principally formed by an increase in particle size in the atmospheric medium, which affects atmospheric absorption, emission, and scattering of light. PM_2.5_: fine inhalable particles, with diameters that are generally 2.5 micrometers and smaller, and originates from construction sites, unpaved roads, fields, smokestacks or fires, including congregated aerosols (e.g. sulfur dioxide, nitrogen dioxide, carbon monoxide, and so on), black carbon (the incomplete combustion of carbonaceous combustibles) [1], dust, sea salt [2], heavy metals, and polycyclic aromatic hydrocarbon [3]. Haze incidents are a relatively new threat to human health [4], air quality [5], global climate change [6], ecological suitability for human settlement, and regional sustainable development.

Recently, haze has become a principal environmental issue in China. Consequently, the causes of particulate pollution have been discussed widely: e.g., secondary aerosol [5], aerosol optical properties [7], and aerosol chemical components [8]. And the formation and evolution mechanism of haze has been similarly explored [9]: e.g., long-lasting haze occurrences in Nanjing [10], a winter regional haze in the North China Plain [11], and the heavy haze pollution episode over central and eastern China [12]. In addition, we know that understanding the origin of fine particulate matter is essential to finding appropriate strategies to combat haze and the harm it causes. Thus, the source apportioning of fine particulate during the haze events in Shanghai [13], Harbin [14], and Fuzhou [15] was implemented, and the characteristics of atmospheric carbonyls were documented [16] in Beijing.

We have known for some time that haze boosts air pollution, causing significant harm to human health [17]. Previous studies have reported extensively on cardiovascular disease, lung disease, exposure time, mortality, and the mechanisms of biochemistry for haze. Short-term exposure was investigated in metropolitan areas [18], and the effects of dust-haze on mortality were explored [19]. In fact, the relationship between haze and respiratory diseases in Brunei Darussalam were analyzed, and it was found that PM_10_ and CO levels have a significant bearing on the incidence of respiratory diseases [20]. In China, Sun et al. [4] explored the relationship between economic development and air pollution, and they found that the variation explained by both total SO_2_ emissions and total smoke and dust emissions were 33 and 24% of pertussis (whooping cough), respectively.

However, source apportioning of fine particulate requires considerable investment in time and money. Thus, in this study, we attempt to analyze source apportioning using data mining. Because the risk of exposure to haze across China has been insufficiently discussed, the object of the present study is to address the relationship between atmospheric contamination and human health in China. Specifically, we seek to accomplish the following: (1) to analyze the spatial-temporal distribution of atmospheric contamination over China; (2) to explore the source apportioning of fine particulate in China; and (3) to analyze the relationship between atmospheric contamination and the risk of human exposure in China.

## Methods

### Data collection

**Fig. 1:**
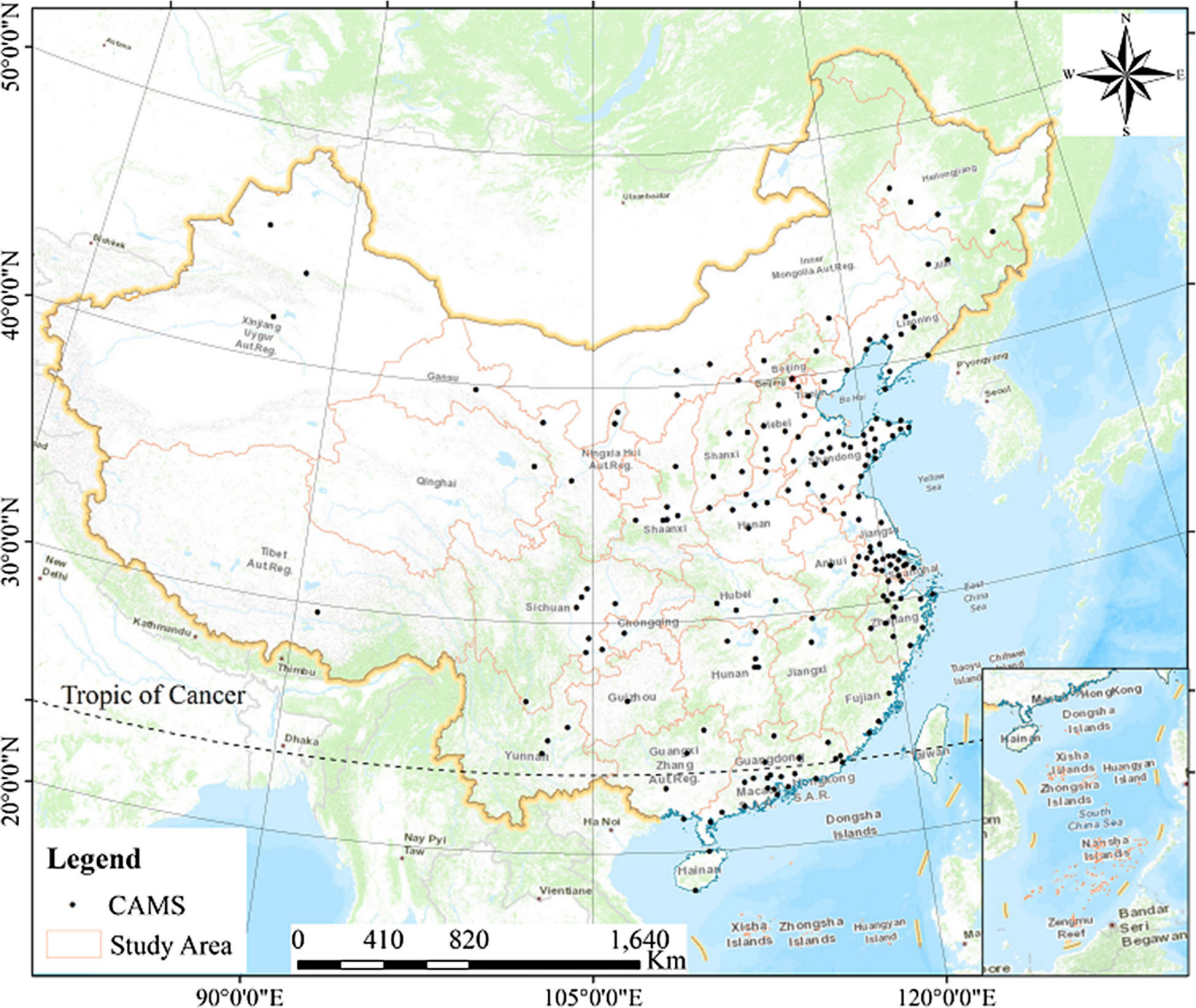
Study areas and the continuous air monitoring stations (CAMS) in China

All air measurements (SO_2_, NO_2_, CO, O_3_, PM_10,_ and PM_2.5_) were obtained from 188 main cities with continuous air-monitoring stations (Fig. 1), the stations were set up accord with the standard “Technical regulation for selection of ambient air quality monitoring stations (HJ 664–2013)”, and were obtained from the Ministry of Environmental Protection of the People’s Republic of China (http://datacenter.mep.gov.cn/). The disease data of pertussis was collected from *China Statistical Yearbook* (www.stats.gov.cn, 2004–2015) and *China Statistical Yearbook of Health and Family Planning* (www.moh.gov.cn, 2004–2015). In addition, the atmospheric contamination was compiled annual means to analyze the spatial-temporal distribution of atmospheric contamination over China, and monthly in every city to analyze the relationships among air pollutions for 2014 and 2015.

### Method of health risk assessment

This study used the risk assessment method of the U.S. Environmental Protection Agency (EPA) [21], which focused on the health risk assessment through inhalation pathway for three kinds of people (adult males, adult females, and children), thus, avoided the effects of population density. The assessment study focuses on the risk of exposure to air pollutants (PM_10_, SO_2,_ and NO_2_) in China; *R*_*i*_ was the individual health risk for exposure pollution, calculated as Eq. 1 [22]:

1 $$ {R}_i = AD{D}_{opt}\times {10}^{-6}/\ \left( Rf{d}_{i j}\times 70\right) $$

*ADD*_*opt*_ was the average daily dose, calculated as Eq. 2 [23]:

2 $$ AD{D}_{opt} = \left( CA \times IR \times ED\right)\ /\ \left( BW \times AT\right) $$

where CA was the concentration (mg m^−3^) of air pollutants, the average values of inhalation rate (IR), ED (exposure duration in days) and AT (averaging exposure time in days) were showed in Table 1 [22], and the average weight (BW) was obtained from national physical fitness test communiqués (http://www.gov.cn/test). The Rfd_ij_ (reference dose) values for PM_10_, SO_2,_ and NO_2_ referred to the U.S EPA (https://www3.epa.gov/).

**Table 1:** Parameters for health risk assessment through inhalation pathway

Crowds	IR(m^3^/d)	BW(kg)	ED(d)	AT(d)	CA-PM_10_(mg m^−3^)	CA-SO_2_(mg m^−3^)	CA-NO_2_(mg m^−3^)
Adult male	15.2	60	30 × 365	30 × 365	0.15 (National third standard)	0.06 (National second standard)	0.04 (National first standard)
Adult female	11.3	57	30 × 365	30 × 365			
Children	8.7	44	18 × 365	18 × 365			

Based on the IDW (inverse distance weighted) interpolation method to model the spatial distribution of health risk in China, then, calculated the Rfd_ij_ values and reclassified by the national air quality standard to get the expose risk level of air pollutants.

### Tools of analysis

In the present study, the ArcGIS 10.2 (ESRI, Inc., Redlands, CA, USA) was used to draw spatial graphs, and SigmaPlot for Windows 10.0 (Systat Software, Inc., Chicago, IL, USA) was used to conduct correlation and regression analysis. Correlations between different variables were determined using two-tailed Pearson’s Correlation at 0.05 levels.

## Results

### The size of main air pollutants

**Fig. 2:**
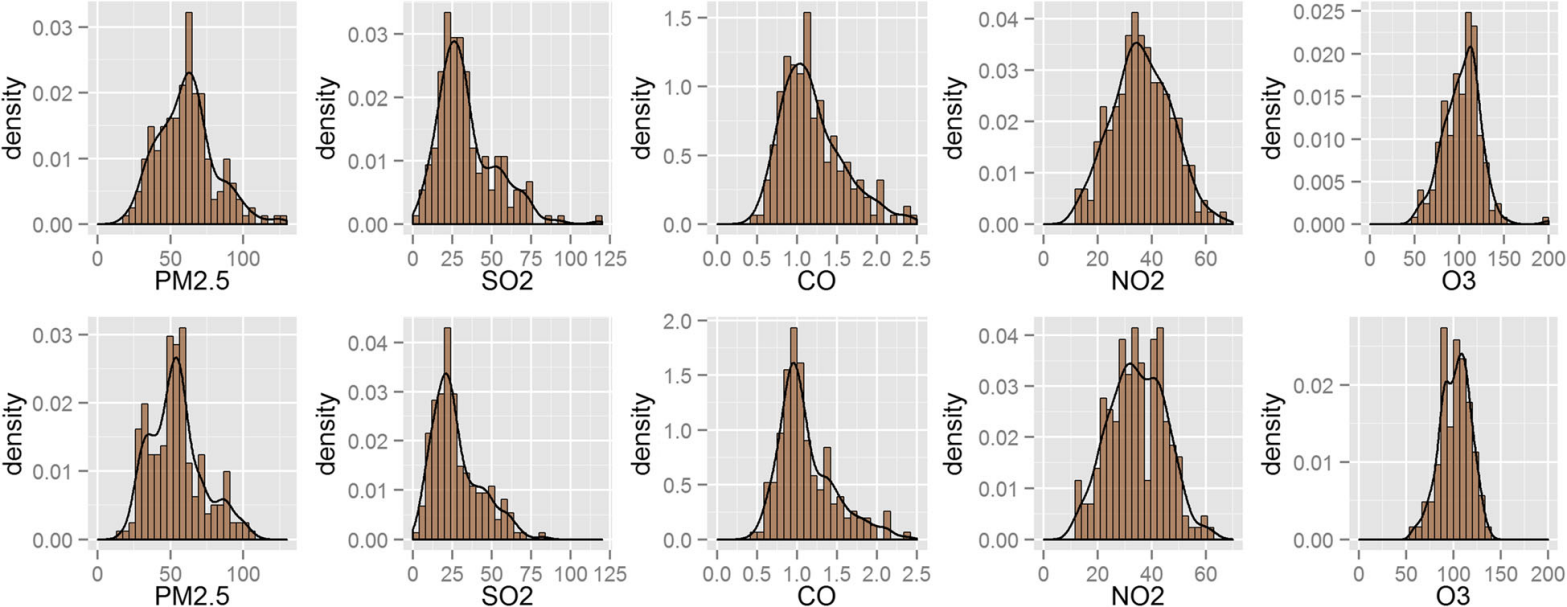
The frequency distribution of the principal air pollutants. The upper and blow panels represent the documents were collected in 2014 and 2015

As shown in Fig. 2, the frequency distribution of air pollutant (PM_2.5_, SO_2_, CO, NO_2,_ and O_3_) concentrations in 2014 and 2015 was observed. The values of the PM_2.5_, SO_2_, CO, NO_2_ and O_3_ range from 18.58 μg m^−3^ to 130.46 μg m^−3^, from 2.17 μg m^−3^ to 117.82 μg m^−3^, from 0.47 μg m^−3^ to 2.42 mg m^−3^, from 12.56 μg m^−3^ to 66.09 μg m^−3^, from 48.78 μg m^−3^ to 198.61 μg m^−3^, with median values of 61.44 μg m^−3^, 30.11 μg m^−3^, 1.12 μg m^−3^, 36.05 μg m^−3^, 103.97 μg m^−3^, respectively. The high occurrence frequency of the PM_2.5_, SO_2_, CO, NO_2,_ and O_3_ was around 40–75 μg m^−3^, around 15–40 μg m^−3^, around 0.75–1.3 mg m^−3^, around 25–50 μg m^−3^, and around 75–130 μg m^−3^ (upper panel). In 2015, there was not only a similar shift in the trend of air pollutants (PM_2.5_, SO_2_, CO, NO_2,_ and O_3_) observed but also the median concentration of air pollutants reduced and centered generally in 52.84 μg m^−3^, 23.52 μg m^−3^, 1.02 mg m^−3^, 34.18 μg m^−3^, 102.67 μg m^−3^, respectively. As an example, the values of the PM_2.5_, SO_2_, CO, NO_2_ and O_3_ range from 17.05 to 106.32 μg m^−3^, from 2.89 to 82.05 μg m^−3^, from 0.44 to 2.36 mg m^−3^, from 12.84 to 61.24 μg m^−3^, from 58.56 to 136.38 μg m^−3^. In addition, the high occurrence frequency of the PM_2.5_, SO_2_, CO, NO_2_ and O_3_ narrowed and became more centralized at the range of 25–55 μg m^–3^, around 10–30 μg m^−3^, around 0.75–1.25 mg m^−3^, around 25–50 μg m^−3^, and around 90–125 μg m^−3^ (below panel), respectively.

### Relationships among principal air pollutants over China from 2014 to 2015

**Fig. 3:**
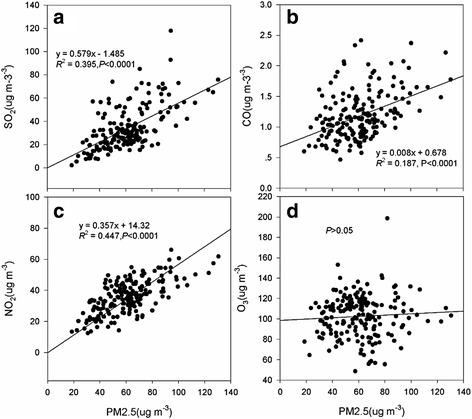
The PM_2.5_ was explained by the principal air pollutants over China (2014), and graph **a**, **b**, **c**, and **d** represent SO_2_, CO, NO_2_, O_3_, respectively

We postulated that the entire concentration of PM_2.5_ depends on air pollutants (SO_2_, CO, NO_2,_ and O_3_), and, per regression analysis, there are close positive correlations between PM_2.5_ and SO_2_, CO, NO_2_ in 2014. In Fig. 3 the appropriate functions of SO_2_, CO and NO_2_ with PM_2.5_ are *Y* = 0.579*X*-1.485 (*R* ^*2*^ = 0.395, *P* < 0.0001) (Fig. 3a), *Y* = 0.008*X* + 0.678 (*R* ^*2*^ = 0.187, *P* < 0.0001) (Fig. 3b), and *Y* = 0.357*X* + 14.32 (*R* ^*2*^ = 0.447, *P* < 0.0001) (Fig. 3c), respectively. However, there was a weak correlation trend between PM_2.5_ and the concentration of O_3_ (*P* > 0.05) (Fig. 3d). Compared to other air pollutants, NO_2_ had the greatest influence on PM_2.5_. Thus, the contribution rate of SO_2_ and NO_2_ is as high as 44.7% for PM_2.5_.

**Fig. 4:**
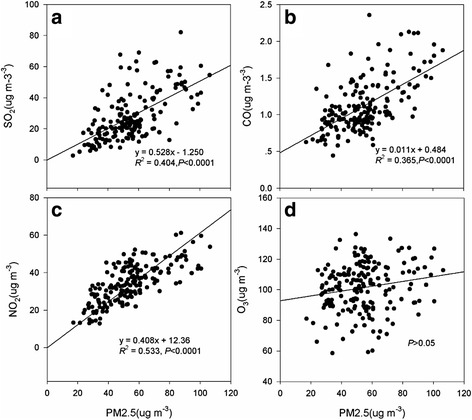
The PM_2.5_ was explained by the principal air pollutants over China (2015), and graph **a**, **b**, **c**, and **d** represent SO_2_, CO, NO_2_, O_3_, respectively

As illustrated in Fig. 4, the concentration of PM_2.5_ had been increasing with the rise of SO_2_, CO, NO_2_, and there are significant relationships between them. High correlation coefficients were noted between SO_2_ (*R* ^*2*^ = 0.404, *P* < 0.0001, Fig. 4a), CO (*R* ^*2*^ = 0.365, *P* < 0.0001, Fig. 4b), NO_2_ (*R* ^*2*^ = 0.533, *P* < 0.0001, Fig. 4c) and PM_2.5,_ which illustrates that the PM_2.5_ are significantly associated with SO_2_, and NO_2_, with CO following, but there were no observable relationships between PM_2.5_ and O_3_ (Fig. 4d).

**Fig. 5:**
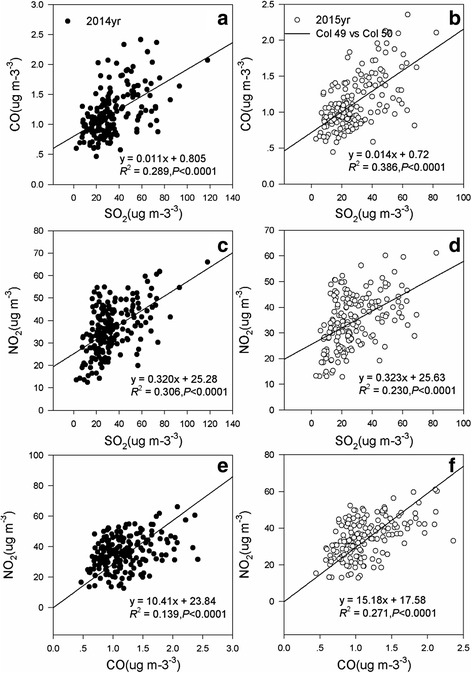
The coupled analysis of the principal air pollutants in China. The graph **a**, **c**, and **e** represent relationships among SO_2_, CO, and NO_2_ in 2014, and the graph **b**, **d**, and **f** represent relationships among SO_2_, CO, and NO_2_ in 2015

Based on regression analysis, we found close relationships among SO_2_, CO and NO_2_ in 2014 and 2015. The appropriate functions were *Y* = 0.011*X* + 0.805 (*R* ^*2*^ = 0.289, *P* < 0.0001) (Fig. 5a), *Y* = 0.320*X* + 25.28 (*R* ^*2*^ = 0.306, *P* < 0.0001) (Fig. 5c), and *Y* = 10.41*X* + 23.84 (*R* ^*2*^ = 0.139, *P* < 0.0001) (Fig. 5e) in 2014. Similar positive correlations were also observed in 2015 with *R* ^*2*^ = 0.386 (*P* < 0.0001, Fig. 5b), *R* ^*2*^ = 0.230 (*P* < 0.0001, Fig. 5d), and *R* ^*2*^ = 0.271 (*P* < 0.0001, Fig. 5f), respectively.

### Spatial patterns of air pollutants in China from 2014 to 2015

**Fig. 6:**
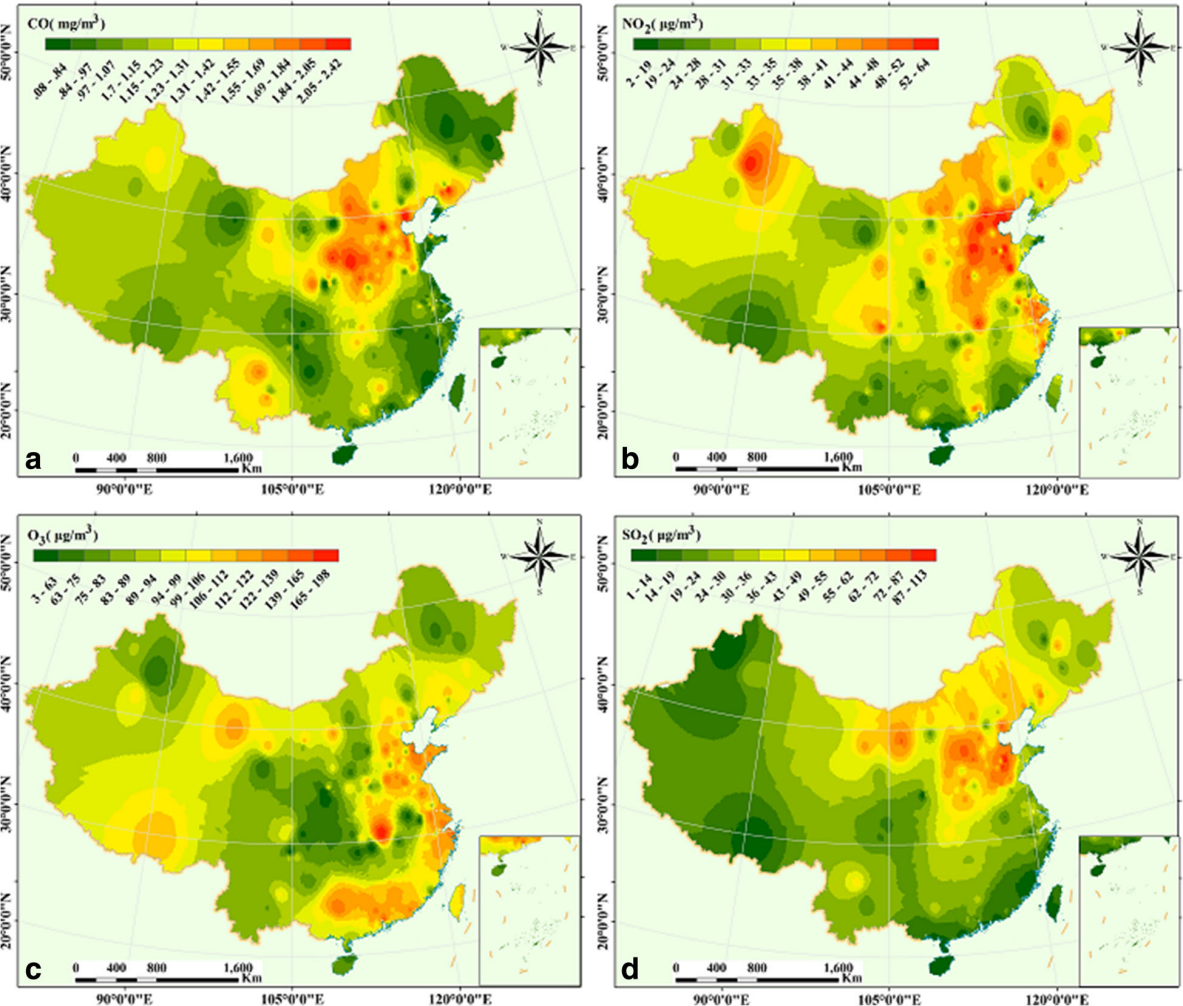
Spatial patterns of air pollutants in China (2014), and the graph **a**, **b**, **c**, and **d** represent CO, O_3_, NO_2_, and SO_2_, respectively

According to the spatial distribution of air pollutants in China in 2014 (Fig. 6), we discovered that the concentration of CO ranges from 0.08 mg m^−3^ to 2.42 mg m^−3^, with the maximum distribution around Hebei and Shanxi province, and the minimum distribution in the southeast, northwest, and northeast (Fig. 6a). In contrast, the concentration range of NO_2_ was between 2 μg m^−3^ and 64 μg m^−3^, with the maximum distribution occurring in Beijing, Tianjin, Hebei, Shandong, Henan province, and northeastern Xinjiang (Fig. 6b). However, O_3_ concentrations ranged from 3 μg m^−3^ to 198 μg m^−3^, with the maximum distribution in eastern China, southern China, and Hubei province; and the minimum distribution regions including Shanxi, Sichuan and Chongqing (Fig. 6c). The concentration of SO_2_ ranges from 1 μg m^−3^ to 113 μg m^−3^, with the maximum distribution in northern China and in Shandong province (Fig. 6).

**Fig. 7:**
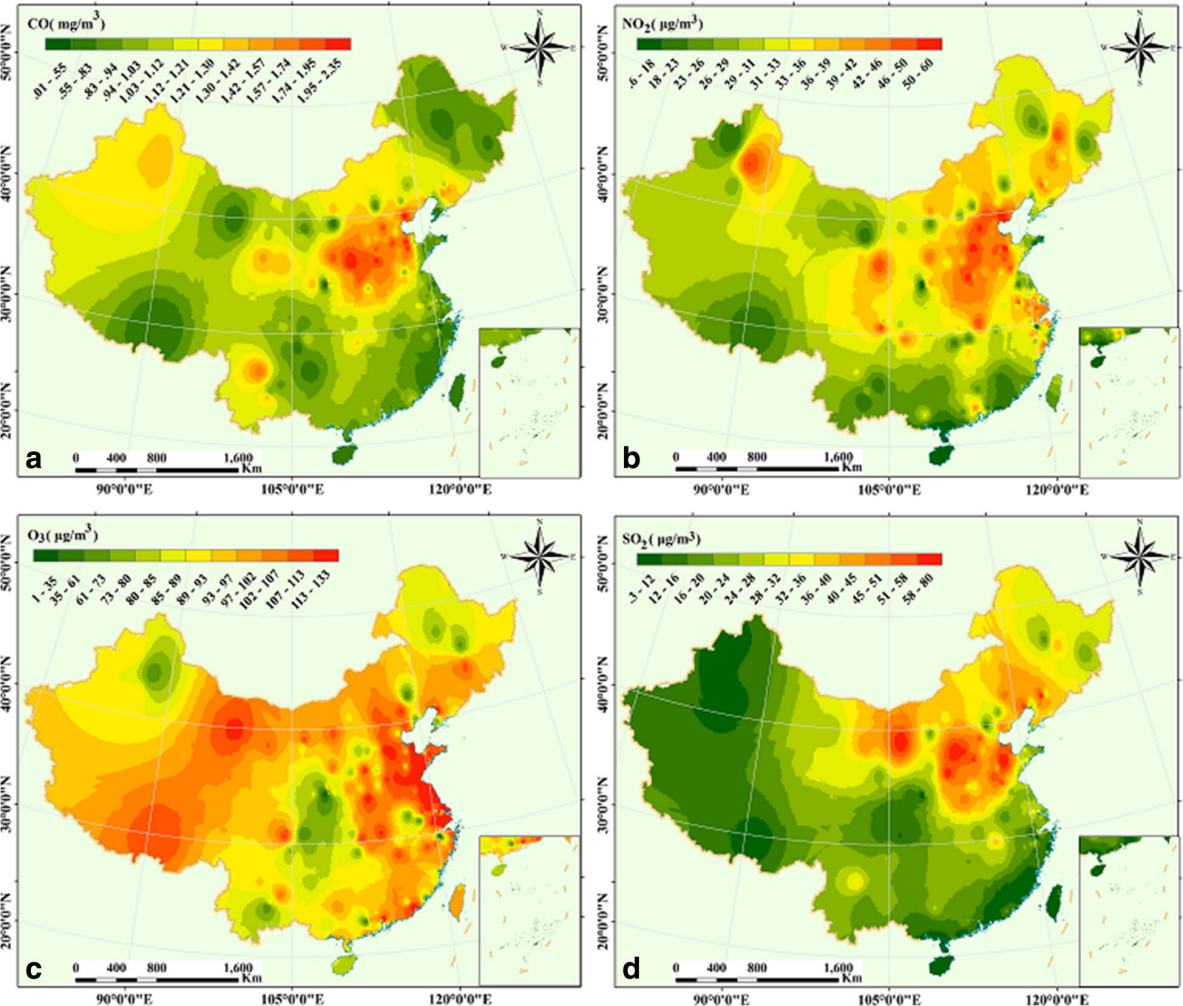
Spatial patterns of air pollutants in China (2014), and the graph **a**, **b**, **c**, and **d** represent CO, O_3_, NO_2_, and SO_2_, respectively

According to Fig. 7a, the concentration of CO ranges from 0.01 mg m^−3^ to 2.35 mg m^−3^ in Shanxi, Shandong, Hebei, Henan, Beijing and Tianjin had the maximum values; we also found that the minimum was chiefly located in Heilongjiang, Gansu and Tibet, and the southeast of China (the coastal urban belt). The concentration ranges of NO_2_ was between 0.6 μg m^−3^ and 60 μg m^−3^, with maximum values distributed primarily in northeastern China (the Beijing-Tianjin-Hebei-Shanxi-Henan-Shandong region, Fig. 7b). In addition, we found that the concentration of O_3_ ranges from 1 μg m^−3^ to133μg m^−3^ in Fig. 7c. The higher concentration values were observed in most areas of China, including eastern China, northern and central China (except Hunan province), and the regions of Gansu, Qinghai, Tibet (around Lhasa) and the Pearl River Delta region. The concentration of NO_2_ ranged from 0.3 μg m^−3^ and 80 μg m^−3^, with maximum values primarily distributed in Shanxi, Shandong and Hebei provinces (Fig. 7d).

**Fig. 8:**
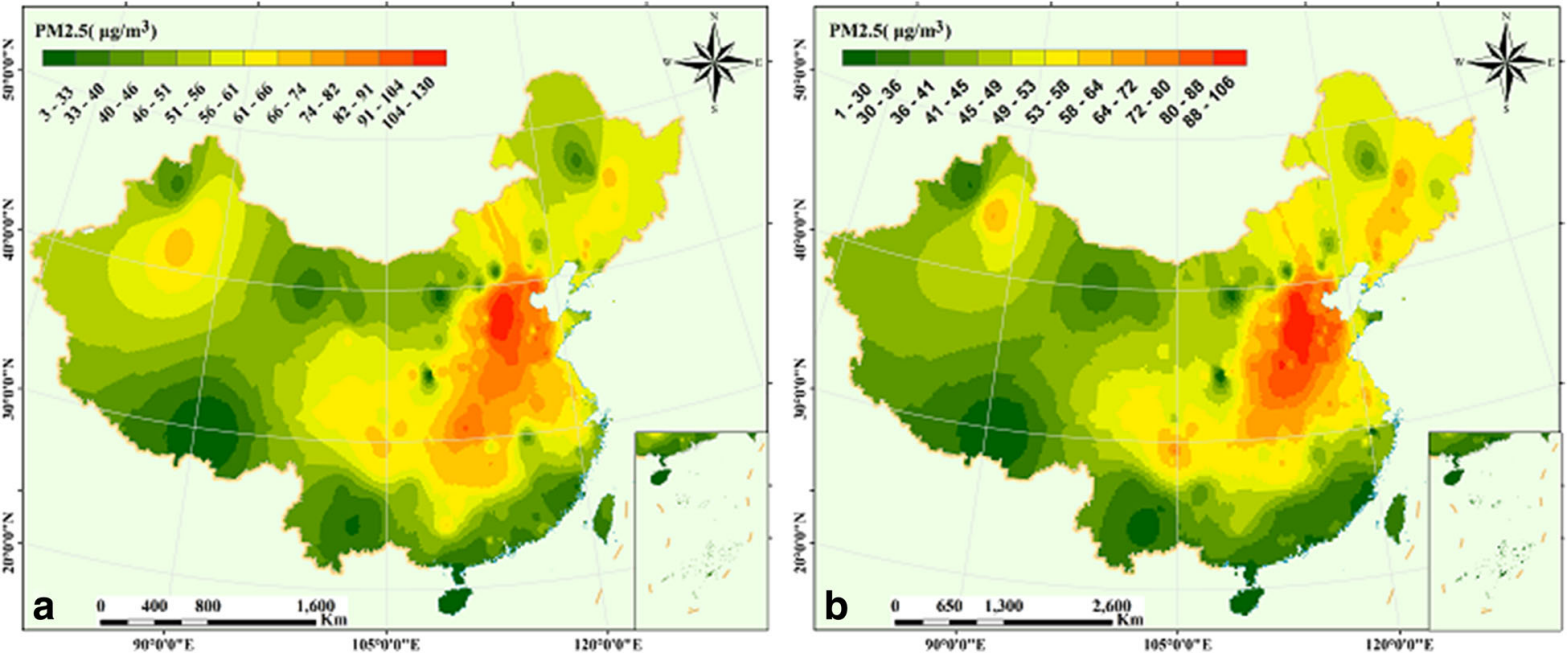
Spatial patterns of air pollutant in China (graph **a**, **b** represent PM_2.5_ in 2014 and 2015, respectively)

The PM_2.5_ concentration ranges from 3 μg m^−3^ to 103 μg m^−3^ in 2014 (Fig. 8a), with the maximum values distributed mainly around Hebei province (Beijing-Tianjin and a part of Shandong-Henan-Hubei. The concentration of PM_2.5_ ranges from 1 μg m^−3^ to 106 μg m^−3^ in 2015 (Fig. 8b), with maximum distribution in Hebei, Shandong, Henan and Hubei provinces, and the region of Central Bohai, while minimum values were observed in areas that include Tibet, Yunnan, Hainan, Fujian, the Pearl River Delta region and the northwest of Gansu province.

### Risk assessment of population exposure to air pollutants in China from 2014 to 2015

**Fig. 9:**
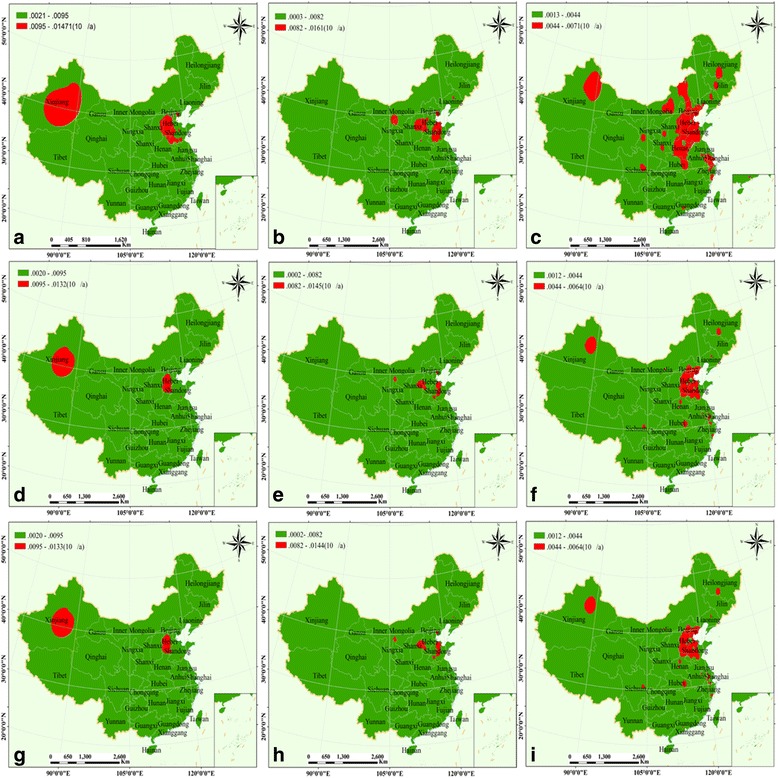
Risk assessment of population exposure to air pollutants in China (2014), and the graph **a**, **b**, **c**/**d**, **e**, **f**/**g**, **h**, **i** represent PM_10_, SO_2,_ and NO_2_ for adult male/adult female/children, respectively

For adult males, according to Fig. 9a, the high-risk values of PM_10_ main distributed in central of Xinjiang province, in Hebei province, the southwest of Shandong and eastern of Shanxi, and a part of Beijing and Tianjin. The high-risk values of SO_2_ occurring mainly in the central of Shandong province, the border of Hebei and Shanxi, a small part of Inner Mongolia (Erdos) and Liaoning provinces (Shenyang) (Fig. 9b). Surprisingly, the high-risk values of NO_2_ were mainly distributed in northeast China (Fig. 9c), regions of Hebei-Shandong-Henan-Beijing-Tianjin, a part of Inner Mongolia, the provincial capital cities of Guangzhou, Chengdu, Lanzhou, Xian, Shenyang, Changchun and Harbin, and the central of Jiangsu province. For adult females and children, a similar distribution pattern of the high-risk values for PM_10_/SO_2_/NO_2_ was observed. The high-risk values for PM_10_ distributed primarily in the central of Inner Mongolia, the south of Hebei province (Fig. 9d and g). The high-risk values for SO_2_ distributed in the central of Shandong province, the border of Shanxi and Hebei province, and a part of Erdos (Fig. 9e and h). As shown in Fig. 9f and i, the high-risk values of distributed primarily in Beijing-Tianjin-Hebei-Shandong regions, a part of Xinjiang province, and the cities of Chengdu, Shanghai, Wuhan, Wenzhou and Harbin.

**Fig. 10:**
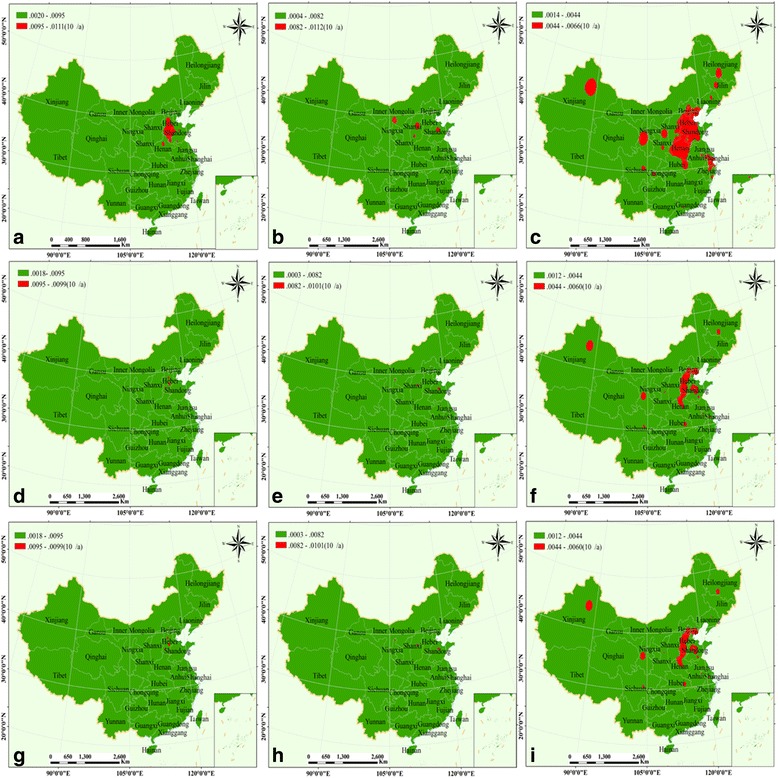
Risk assessment of population exposure to air pollutants in China (2015), and the graph **a**, **b**, **c**/**d**, **e**, **f**/**g**, **h**, **i** represent PM_10_, SO_2,_ and NO_2_ for adult male/adult female/children, respectively

In 2015, for adult males, the high-risk values of PM_10_were mainly distributed around the border of Hebei-Shandong-Henan province, thus, the cities of Baoding, Hengshui, Xingtai, Handan, Shijiazhuang in Hebei; Liaocheng, Dezhou and Heze in Shandong; and Zhengzhou in Henan (Fig. 10a). As shown in Fig. 10b, the high-risk values of SO_2_ occurring in the central of Shanxi (Taiyuan and Linfen) and Shandong province, and a small part of Inner Mongolia (Erdos). The high-risk values of NO_2_ mainly distributed in the central and northeastern of China (Fig. 10c), regions of Beijing–Tianjin–Hebei-Shandong-Henan, the central of Jiangsu province, and the cities of Urumchi, Lanzhou, Yanan, Chengdu, Shenyang, Changchun and Harbin, and the main city area of Chongqing. As for adult females and children, the regions of high-risk values for PM_10_/SO_2_/NO_2_ were alike. As for PM_10_, the high-risk values distribution primarily in the main city area of Baoding, Hengshui, and Handan (Fig. 10d and g). The high-risk values for SO_2_ occurring in the central of Shandong, the main city area of Taiyuan and Shizuishan (Fig. 10e and h). As shown in Fig. 10f and i, the high-risk values of NO_2_ were chiefly distributed in Beijing-Tianjin-Hebei regions, the central of Shandong and the north of Henan province; and the province capital cities of Urumchi, Lanzhou, Chengdu, Wuhan and Harbin.

### Relationships between air pollution and human health

**Fig. 11:**
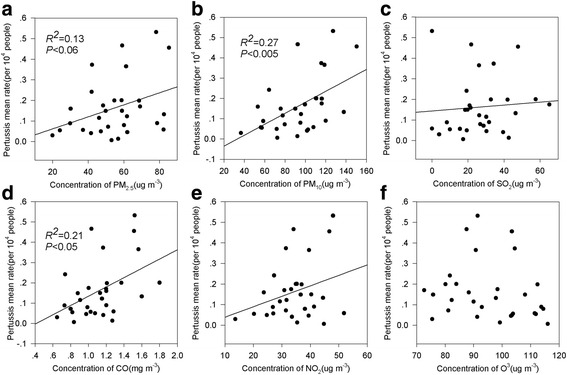
The relationships of pertussis mean rate and the average concentration of air pollutions from 2014 to 2015 in 30 provinces. The graph **a**, **b**, **c**, **d**, **e** and **f** represent the relationship between pertussis mean rate and PM_2.5_, PM_10_, SO_2_, CO, NO_2,_ and O_3_

The concentrations of air pollutions were the average values during 2014–2015, the mean rate of total pertussis was calculated from 30 provinces from 2004 to 2014. General linear models analysis illustrated that the mean rate of total pertussis was significantly associated with the average concentrations of PM_2.5_, PM_10_ and CO (Fig. 11a, b, and d), and the variation explained by them were 61% (*P* < 0.06) for the rate of total pertussis. Meanwhile, the rate of total pertussis was related to SO_2_ and NO_2_ to some extent (Fig. 11c and e). However, there was no significant relationship between O_3_ and total pertussis (Fig. 11f).

## Discussion

### Relationships among air pollutants

For this study, the most extensive data for analyzing the concentration and relationship of air pollutants and time-series datasets in China were used, with the aim of understanding pollution and to mitigate the heavy haze on the Chinese mainland. The Min, Median and Max concentration of air pollutants in 2014 and 2015 were presented, after finding that the occurrence frequency narrowed and became more centralized from 2014 to 2015 (Fig. 2). Our study also identified that the main components were NO_2_ and SO_2_ in PM_2.5_ (*R* ^*2*^ = 0.395, *R* ^*2*^ = 0.447) in 2014 (Fig. 3) and (*R* ^*2*^ = 0.404, *R* ^*2*^ = 0.533) in 2015 (Fig. 4) based on regression analysis. The same phenomenon was found in Beijing [9], central and eastern China [12, 24, 25]. Previous research has observed that the concentration of SO_2_ and NO_2_ is lowest in the autumn and highest in the winter [26]. The concentration of PM_2.5_ is closely correlated with SO_2_ and NO_2_ in Xi’an [23]. A joint effect of NO_2_ and PM was found [27]. Wang not only analyzed the relationships of NO_2_, SO_2,_ and PM_2.5_ but also found that with the increase of sulfate and nitrate, their particle hygroscopicity enhances and drives the formation and evolution of haze pollution [12]. This means that SO_2_ and NO_2_ play an important role in the enhancement of PM_2.5_ [28], and reminds us to explore the relationships between SO_2_, NO_2,_ and CO.

As shown in Fig. 5, the most significant correlation coefficient was NO_2_ and SO_2_ (*R* ^*2*^ = 0.306), followed by CO and SO_2_ (*R* ^*2*^ = 0.289); NO_2_ and CO (*R* ^*2*^ = 0.139) were the least pronounced in 2014. Nevertheless, a different significant association order was observed in 2015. Thus, the correlation coefficient of CO and SO_2_ (*R* ^*2*^ = 0.386) was greater than NO_2_ and CO (*R* ^*2*^ = 0.271) in 2015. This result suggests a strong relationship between CO and SO_2_, and NO_2_ and CO; one study reported that the critical factor for formation droplet-mode particles was the availability of the water-vapor contents and precursor gases (SO_2_ and NO_2_) [29]; in other words, under iron and manganese catalysis, the heterogeneous oxidation of SO_2_ and NO_2_ change into the secondary sulfates (SO_4_^2−^ and NO_3_^−^) in the droplet mode [30, 31], namely, the complex interaction of SO_4_^2−^, NO_3_^−^, congregated aerosols (e.g. sulfur dioxide, nitrogen dioxide, carbon monoxide, and so on), black carbon (the incomplete combustion of carbonaceous combustibles) determined the formation of haze and its particulate size [1, 29, 32]. NH_3,_ however, should not be neglected, which may result in the particulate sulfate and nitrate increase [28].

### The spatial patterns of air pollutants

Apart from the regression analysis of air pollutants, spatial patterns of yearly average simulation values clearly present different air pollutants concentration distributions in different regions in 2014 and 2015 (Figs. 6 and 7). A few studies found that the Beijing–Tianjin–Hebei-Shandong-Shanxi-Henan regions had the highest concentration of air pollutants [24, 25], including the local characteristics of high populations, city traffic, exhaust emissions, and rapid urban expansion [23, 33]. In this study, the same spatial patterns of air pollutants were observed (Figs. 6, 7 and 8). Furthermore, we found the concentration of SO_2_, NO_2,_ and CO in Inner Mongolia cannot be negligible (Fig. 6a, b and d); meanwhile, the Tibet Plateau and coastal areas from Tianjin to Guangxi were affected by O_3_ (Fig. 6c) in 2014. In addition, similar spatial patterns of the maximum values were observed in China. However, from 2014 to 2015, the spatial variation of O_3_ concentration displayed a rapid increasing trend in China, especially on the Tibet Plateau (Fig. 7c), which may be influenced by its origin and long-distance transport. The PM_2.5_ is distributed mainly in the region of Beijing-Tianjin-Shandong-Henan-Hubei in 2014 and 2015. Fortunately, its distribution narrowed and became more centralized in2015, and showed that the extent and area of PM_2.5_ were lower than in 2014.

Emissions of PM_2.5_ (97%), SO_2_ (90%), NO_2_ (70%) and CO (32%) were mainly due to the combustion of coal [34]. A number of previous studies have explored the origin and transportation [32, 35] of air pollutants [36, 37], including the congregated aerosols (e.g. sulfur dioxide, nitrogen dioxide, carbon monoxide, and so on), black carbon (the incomplete combustion of carbonaceous combustibles) [17], dust, sea salt [2], heavy metal, and polycyclic aromatic hydrocarbon [3], which resulted from vehicle exhaust [36], coal consumption, secondary production, stagnant meteorological conditions [13, 38–40], biomass open burning [41, 42], and petrol stations [43]. Therefore, replacing coal and fossil fuels with cleaner fuels were the fundamental methods of controlling the concentration of air pollutants [39]. Certainly, we need to encourage new technologies and energy sources for automobiles, still a major contributor to air pollution [44]. But uncertainties exist from various sources, particularly the air pollutants in Xinjiang Uygur Autonomous Region, which might cause sand-dust storms. Therefore, ecological conservation projects should be developed and implemented; for instance, building key forest shelterbelts to shield against sandstorms in Xinjiang.

### Health risk assessment and human health

*R*_*i*_ for adult males, adult females, and children, obtained for different pollutants (PM_10_, SO_2,_ and NO_2_) in 2014 and 2015. It was observed that the high-risk of PM_10_was mainly distributed in the cities of Baoding, Hengshui, Xingtai, Handan, Shijiazhuang, Liaocheng, Dezhou, Heze and Zhengzhou (Figs. 9a, d, g and 10a, d, g), and Urumchi (Fig. 9a, d and g). The high-risk values of SO_2_ were chiefly distributed in the cities of Taiyuan and Linfen, a small part of Erdos, and the central of Shandong province (Figs. 9b, e, h and 10b, e, h). The high-risk values of NO_2_ were mainly occurring around in coastal areas from Beijing-Yangtze River Delta region-Pearl River Delta region-central, especially the cities of Urumchi, Lanzhou, Chengdu, Wuhan and Harbin (Figs. 9c, f, i, and 10c, f, i). In comparison, the coverage area of the highest risk level was smaller in 2015 (Figs. 9 and 10).

A large portion of China’s population has been significantly exposed to high-risk areas. Feng et al. [45] evaluated the ILI risk significantly associated with the concentrations of PM in Beijing during the flu season. In Guangzhou, the dust haze clearly increased mortality [19], and the PM_2.5_ contains toxic micro-particles that might increase the risk of respiratory disease [46]. Mortality rates increased due to the high PM pollution in Shenzhen, especially for the elderly and male populations [47]. The cardiovascular, nervous system, respiratory and blood vessels of the brain are damaged by exposure to high concentrations of PM_2.5_ [48]. Indeed, hemorrhagic stroke was closely associated with PM pollution [49]. Lung and cardiovascular disease are related to PM and NO_2_ [50], and NO_2_ was identified as the principal pollutant for respiratory diseases [18]. Local residents in Shanghai were exposed to high health risks due to NO_2_ [26]. Vulnerable people particularly (asthmatics, children, and the elderly), but all people generally, should not be exposed to high concentrations of SO_2_ for any length of time [51]. Meanwhile, high concentrations of O_3_ will irritate the eyes, nose, and throat, although long-term effects, if any, need further research [50]. One researcher has revealed that individuals exposed to biomass burning-impacted aerosols over the long term increased carcinogenic risk [6]. For these reasons and more, it is a matter of considerable urgency that policies be developed and implemented to mitigate the heavy haze in China.

### Limitations of the current study

In this study, although 188 main cities across China used to get the spatial distribution patterns of air pollutions, uncertainties exist for limited data, especially in the sparsely distributed area of Xinjiang, Tibet, and Qinghai. Though significant relationships among main air pollutions were observed, inorganic substance and organic matter also correlated with each other in haze. In addition, PM_2.5_ has other sources of crustal materials, such as from Asian dust storms. We analyzed the relationships among gaseous pollutant emissions. Meanwhile, the average parameter values for crowds in Eq. 2 referred from articles rather than actual measurements, led to the above conclusions about health risk in China. In addition, the data of pertussis was collected from the *China Statistical Yearbook on Environment*, although exposure to pollutions related to the increases in morbidity, accurate and concrete data for long-term effects is urgently needed. Thus, detailed data need collect to define air pollutions and risk assessment of human health in future.

## Conclusions

Air pollution is harmful to the environment and to public health. This study focused on the source apportioning and the spatial-temporal characteristics of air pollutants and analyzed the relationship between atmospheric contamination and human exposure risk in China from 2014 to 2015. The main conclusions are as follows:

1. Regression analysis illustrates that there are close positive correlations between PM_2.5_ and SO_2_, CO and NO_2_, but weak correlations with O_3_ in 2014 and 2015. Additionally, the relationships between SO_2_, NO_2_ and CO were significant, suggesting that vehicle exhaust, coal consumption secondary production, stagnant meteorological conditions, and biomass open-burning are the main factors driving the formation and evolution of air pollution.
2. In general, air pollutants in China have stabilized, showing a slight decline from 2014 to 2015. Accompanying the highest concentrations are high-risk areas distributed in provinces of Hebei, Shanxi, and Henan, and along the coast from Beijing-Yangtze River Delta to the Pearl River Delta region. The high-risk of NO_2_ occurred in the Beijing–Tianjin–Hebei economic belts.

Measuring air pollutants, tracking contaminant paths and assessing pollutants in different areas with volatile weather conditions are complex challenges and need further research. The objective of this study is to help provide healthy, sustainable development not only for the people of China but for developing and developed nations alike. In particular, this study aims to initiate a constructive forum on the Beijing-Tianjin-Hebei collaborative development.
